# Breaking the Binary: Rethinking Sex-Matched Norms in Swallowing Assessments for Gender-Diverse Patients

**DOI:** 10.1177/00034894251338432

**Published:** 2025-06-05

**Authors:** Nina Patel, Rohith M. Bhethanabotla, Sarah L. Schneider, VyVy N. Young, Cara Evans

**Affiliations:** 1 Department of Otolaryngology—Head and Neck Surgery, University of California San Francisco, San Francisco, CA, USA

## Introduction

A videofluoroscopic swallow study (VFSS) is an imaging test used to evaluate swallowing function, first described by Logemann et al^1^ Dynamic swallow study (DSS) provides objective kinematic data to assess swallowing disorders, measuring pharyngeal and laryngeal movement at rest and during maximal constriction. Repeated DSS measures allow for a more objective method of tracking swallowing changes over time due to treatment or disease progression.

Clinicians typically interpret pharyngeal kinematic data using sex- and age-matched norms, given established anatomical differences. Leonard et al^2^ and Kendall et al^3^ identified sex-based differences in swallowing measures, except for pharyngoesophageal sphincter opening (PESmax). Normative data, first established by Leonard et al,^2^ define abnormal variation as >2 standard deviations (SD) from male or female norms, though other methodologies use different cutoff points. For instance, the Swallowtail program (Belldev Medical) defines norms as 1 SD below the group mean, while Jardine et al^4^ relies on interquartile ranges. ^5^ Table 1 summarizes key measures used in swallow kinematics and findings in the literature regarding potential sex differences.

**Table 1.** Pharyngeal Swallowing Kinematics: Key Measures and Findings.

Measure	Definition	Findings from the literature
Hyoid bone elevation and excursion		
Hant	Distance the hyoid bone travels anteriorly: (HAntMax-HAntRest)	Increases with bolus volume; greater in males than females^6^
Hsup	Distance the hyoid bone travels superiorly: (HSupMax-HSupRest)	Increases with bolus volume; greater in males than females^6^
Hmax	Overall distance the hyoid travels from hold position to maximal elevation/excursion	Hyoid elevation increases with bolus volume; greater in males than females; displacement related to height^7,8^
Hyoid to larynx approximation		
HLhold	Distance between the lowest portion of the hyoid and the superior anterior border of the trachea at rest	Larger in males than females^7^
HLapprox	Distance between the same 2 points at maximum approximation during swallow	Larger in males than females^7^
HL	Difference between HLhold and HLapprox	Larger in males; no correlation with height, weight, or BMI^7^
HL + Hmax	Sum of HL and Hmax representing overall laryngeal complex movement	Influenced by sex differences in HL and Hmax; used to evaluate laryngeal functionality for airway safety^7^
Pharyngoesophageal Sphincter (PES) Opening Size	PESmax: Narrowest point in PES opening (between C3 and C6)	Increases with bolus volume; no sex differences^9^
Pharyngeal area		
PAH	Pharyngeal area at bolus hold position (lateral view)	Males have a slightly larger mean pharyngeal hold area and a longer pharynx than females^10^
PAM	Pharyngeal area at maximal constriction during 20-cc bolus swallow	Strongest influencing factor is sex, though age and BMI may contribute^10^
Pharyngeal Constriction Ratio (PCR)	Ratio of PAmax (max pharyngeal area) to PAhold (area at bolus hold)	Larger with increasing bolus size and in males; no correlation with height, weight, or BMI^7,11^

Furthermore, hyoid displacement, hyoid-to-larynx approximation, and PAmax/PAhold differed significantly for males and females. While use of sex-based normative values can be appropriate for cisgender patients, its use for gender-diverse individuals, particularly those who have undergone gender-affirming hormone treatment (GAHT), may not be as straightforward. The timing of GAHT relative to puberty adds further complexity. A study by Willemsen et al^12^ found that transgender boys receiving testosterone therapy reached a taller adult height than predicted, particularly for those who started treatment earlier. Alternatively, Boogers et al^13^ showed a significant decrease in adult height in transgender girls taking ethinyl estradiol. These findings indicate that GAHT can influence linear growth and adult height, depending on when GAHT begins (ie, either pre- or post-puberty).

The population of transgender and gender-diverse individuals, though historically small, is rapidly growing, reflecting broader social and cultural shifts toward greater recognition and acceptance of gender diversity. As societal awareness increases and more individuals seek gender-affirming care, the healthcare system must adapt to meet the unique needs of this population. One area where this adaptation is particularly important is in the assessment and treatment of swallowing physiology. Given that clinicians have traditionally relied on sex-based norms to evaluate swallowing function, this can present significant challenges when applied to gender-diverse patients whose physiology may not align with these norms. For transgender and nonbinary individuals, who may undergo GAHT or gender-affirming surgeries, these traditional sex-based benchmarks may not accurately reflect their physiological realities. To date, no studies have directly questioned the applicability or validity of binary sex-matched pharyngeal kinematic norms for assessing swallowing function in gender-diverse populations. Given that up to 1% of the U.S. population identifies as gender-diverse and numbers are rapidly increasing, this poses a challenge to speech-language pathologists (SLPs) who must decide whether to use sex-based normative values to evaluate swallow function for the individual patient.^14^ This piece hopes to initiate a conversation and bring awareness to how sex-based norms can possibly skew interpretation and understanding of swallow function in gender-diverse individuals.

## Methods

After performing a search through the Head and Neck Surgical Oncology Clinic database for a single tertiary care center, the research team identified 3 openly gender diverse (2 transgender and 1 non-binary) patients seen by SLP with symptoms of dysphagia. A retrospective chart review was conducted to extract sex-normative swallow data, VFSS information, and the early management of their dysphagia diagnosis. All 3 of these patients received at least 1 VFSS. Cisgender patients and those with no VFSS were excluded from this study. Additionally, all data was de-identified and the study received approved from the University of California, San Francisco (UCSF) Institutional Review Board (study #19-29435) to ensure ethical handling of patient data. For data analysis, Leonard et al^2^ based normative data was utilized. Additionally, the Dynamic Imaging Grade of Swallowing Toxicity (DIGEST) was utilized as a standardized scoring system used to grade pharyngeal dysphagia severity based on patterns of penetration/aspiration and pharyngeal residue. DIGEST provides a summary grade ranging from 0 (no impairment) to 4 (life threatening/profound impairment).^15,16^

### Case Study 1

A 53-year-old transgender woman presented with postprandial discomfort, an inability to eruct, and a “croaking” sound after eating. She had been on gender-affirming hormone therapy (GAHT) for 2 years. Given her gender identity, the appropriateness of sex-based VFSS norms was questioned. This patient’s swallow kinematics are summarized in Table 2.

**Table 2.** Case Study 1 Swallowing Kinematics Measures for 1 Patient Videofluoroscopic Swallow Studies (VFSSs) With Reference Values for Male and Female Norms Under the Age of 65 y and Corresponding Standard Deviations.

Patient 1 VFSS 1	Displacement measure	Pt values	Male norms	SD	Female norms	SD
	PAM (cm^2^)^a^	0.48	<0.47	Within 1-1.5SD (0.48-0.57)	<0.34	>2SD Above Mean (>0.50)
	PAH (cm^2^)^b^	10.89	<10	Within 1-1.5SD (10-11.1)	<8.2	>2SD Above Mean (>9.9)
	PCR (cm^2^)^a^	0.04	<0.12	Normal (<0.12)	<0.06	Normal (<0.06)
	HLHold (mm)^b^	37.58	<43.3	Normal (<43.3)	<35.4	Within 1.5-2SD (38.3-41.3)
	Hant (mm)^a^	5.46	>4.03	1-1.5SD Below Mean (4.03-1.87)	>2.85	Normal (>2.85)
	Hsup (mm)^a^	25.86	>16.92	>2SD Below Mean (<10.17)	>11.36	Within 1.5-2SD (8.38-5.41)
	HL (mm)^a^	11.96	>8.4	>2SD Below Mean (<4.3)	>5.2	Within 1-1.5SD (5.2-2.4)
	Hmax (mm)^a^	26.43	>17.2	>2SD Below Mean (<10.04)	>10.08	1-1.5SD Below Mean (10.08-7.15)
	HL + Hmax (mm)^a^	38.39	>28.1	Normal (>28.1)	>24.5	Normal (>24.5)

Abbreviations: Hant, anterior hyoid movement; HL, hyoid-to-larynx approximation; HLhold, hyoid-to-larynx approximation at hold; Hmax, maximal hyoid movement; Hsup, superior hyoid movement; PAM, pharyngeal area at maximum constriction; PAH, pharyngeal area at hold; PCR, pharyngeal constriction ratio.

a Indicates values representing 1 cc volume. ^b^Indicates values 20 cc volume.

VFSS revealed mild pharyngeal dysphagia (DIGEST: 1, Safety: 0, Efficiency: 1, and PAS: 1). Her PAH was >2 SD above female norms and 1 to 1.5 SD above male norms, with male norms considering it normal. PAM was 1.5 to 2 SD below female norms and 1 SD below male norms, leading female norms to suggest mild pharyngeal weakness. PCR was within normal limits for both, though male norms had a broader mean threshold for normal. HLhold was 1 to 1.5 SD above female norms but normal for male norms, highlighting anatomical variations in pharyngeal kinematics.

These findings emphasize that anatomical differences in PAH, PAM, and HLhold, as well as kinematic measures, vary based on sex norms. Using female norms could result in identifying abnormalities that male norms would not, impacting clinical interpretation.

### Case Study 2

A 48-year-old transgender woman with metastatic papillary thyroid cancer underwent total thyroidectomy, median sternotomy, and radiation therapy. The patient began her transition after puberty and has intermittently received GAHT for 30 years. Pre-radiation VFSS was performed as part of a dysphagia pathway (DIGEST: 1, Safety: 1, Efficiency: 0, and PAS: 6). Table 3 highlights the VFSS findings for this individual.

**Table 3.** Case Study 2 Swallowing Kinematics Measures for 2 Patient Videofluoroscopic Swallow Studies (VFSSs) With Reference Values for Male and Female Norms Under the Age of 65 y and Corresponding Standard Deviations.

Patient 2 VFSS 1	Displacement measure	Pt values	Male norms	SD	Female norms	SD
	PAM (cm^2^)^a^	0.61	<0.47	Within 1.5-2SD (0.57-0.66)	<0.34	>2SD Above Mean (>0.50)
	PAH (cm^2^)^b^	10.94	<10	Within 1-1.5SD (10-11.1)	<8.2	>2SD Above Mean (>9.9)
	PCR (cm^2^)^a^	0.06	<0.12	Normal (<0.12)	<0.06	Normal (<0.06)
	HLHold (mm)^b^	39.62	<43.3	Normal (<43.3)	<35.4	Within 1.5-2SD (38.3-41.3)
	Hant (mm)^a^	3.42	>4.03	1-1.5SD Below Mean (4.03-1.87)	>2.85	Normal (>2.85)
	Hsup (mm)^a^	7.63	>16.92	>2SD Below Mean (<10.17)	>11.36	Within 1.5-2SD (8.38-5.41)
	HL (mm)^a^	3.93	>8.4	>2SD Below Mean (<4.3)	>5.2	Within 1-1.5SD (5.2-2.4)
	Hmax (mm)^a^	8.36	>17.2	>2SD Below Mean (<10.04)	>10.08	1-1.5SD Below Mean (10.08-7.15)
	HL + Hmax (mm)^a^	12.29	>28.1	>2SD Below Mean (<19.0)	>24.5	>2SD Below Mean (<20.2)
Patient 2 VFSS 2						
	PAM (cm^2^)^a^	4.2	<0.47	>2SD Above Mean (>0.66)	<0.34	>2SD Above Mean (>0.50)
	PAH (cm^2^)^b^	9.55	<10	Normal (<10)	<8.2	Within 1.5-2SD (9-9.9)
	PCR (cm^2^)^a^	0.44	<0.12	>2SD Above Mean (>0.18)	<0.06	>2SD Above Mean (>0.09)
	HLHold (mm)^b^	48.33	<43.3	Within 1.5-2SD (46.0-48.7)	<35.4	>2SD Above Mean (>41.3)
	Hant (mm)^a^	20.56	>4.03	Normal (>4.03)	>2.85	Normal (>2.85)
	Hsup (mm)^a^	-8.98	>16.92	>2SD Below Mean (<10.17)	>11.36	>2SD Below Mean (<5.41)
	HL (mm)^a^	10.55	>8.4	Normal (>8.4)	>5.2	Normal (>5.2)
	Hmax (mm)^a^	22.44	>17.2	Normal (>17.2)	>10.08	Normal (>10.08)
	HL + Hmax (mm)^a^	32.99	>28.1	Normal (>28.1)	>24.5	Normal (>28.1)

Abbreviations: Hant, anterior hyoid movement; HL, hyoid-to-larynx approximation; HLhold, hyoid-to-larynx approximation at hold; Hmax, maximal hyoid movement; Hsup, superior hyoid movement; PAM, pharyngeal area at maximum constriction; PAH, pharyngeal area at hold; PCR, pharyngeal constriction ratio.

a Indicates values representing 1 cc volume. ^b^Indicates values 20 cc volume.

PAH was 1 to 1.5 SD above male norms (normal range) but >2 SD above female norms, potentially indicating dilation. PAM was 1.5 to 2 SD above male norms and >2 SD above female norms, with female norms suggesting reduced pharyngeal constriction. HLhold was 1.5 to 2 SD above female norms but normal using male norms. Other kinematics (Hant, Hsup, and Hmax) were more sensitive to deficits using male norms, particularly in hyolaryngeal elevation and excursion.

A second VFSS post-radiotherapy (2 years later) showed improvement in PAH, shifting from >2 SD to 1.5 to 2 SD above female norms and from 1 to 1.5 SD above male norms to normal. PAM remained >2 SD in female norms but changed from 1.5 to 2 SD to >2 SD in male norms, better reflecting its significance. PCR worsened in both norms but was more clearly distinguished using male norms. Hyolaryngeal measures showed improvement but varied in SD changes based on gender norms. These results suggest that female norms may lack sensitivity in detecting some kinematic changes.

### Case Study 3

A 29-year-old genderqueer non-binary patient, assigned female at birth, underwent glossectomy, base of tongue resection, free flap reconstruction, radiation, and chemotherapy. No information was collected on GAHT use at that time. A VFSS performed 3 months post-treatment (DIGEST: 0, S: 0, E: 0, and PAS: 2) revealed discrepancies in sex-based norms. A summary of this individual’s swallow kinematics are summarized in Table 4.

**Table 4.** Case Study 3 Swallowing Kinematics Measures for 2 Patient Videofluoroscopic Swallow Studies (VFSSs) With Reference Values for Male and Female Norms Under the Age of 65 y and Corresponding Standard Deviations.

Patient 3 VFSS 1	Displacement measure	Pt values	Male norms	SD	Female norms	SD
	PAM (cm^2^)^a^	0.78	<0.47	>2SD Above Mean (>0.66)	<0.34	>2SD Above Mean (>0.50)
	PAH (cm^2^)^b^	9.23	<10	Normal (<10)	<8.2	Within 1.5-2SD (9-9.9)
	PCR (cm^2^)^a^	0.08	<0.12	Normal (<0.12)	<0.06	Within 1.5-2SD (0.07-0.9)
	HLHold (mm)^b^	26.23	<43.3	Normal (<43.3)	<35.4	Normal (<35.4)
	Hant (mm)^a^	8.69	>4.03	Normal (>4.03)	>2.85	Normal (>2.85)
	Hsup (mm)^a^	3.6	>16.92	>2SD Below Mean (<10.17)	>11.36	>2SD Below Mean (<5.41)
	HL (mm)^a^	3.63	>8.4	>2SD Below Mean (<4.3)	>5.2	Within 1-1.5SD (5.2-2.4)
	Hmax (mm)^a^	9.42	>17.2	>2SD Below Mean (<10.04)	>10.08	1-1.5SD Below Mean (10.08-7.15)
	HL + Hmax (mm)^a^	13.05	>28.1	>2SD Below Mean (<19.0)	>24.5	>2SD Below Mean (<20.2)
Patient 3 VFSS 2						
	PAM (cm^2^)^a^	0.51	<0.47	Within 1-1.5SD (0.48-0.57)	<0.34	>2SD Above Mean (>0.50)
	PAH (cm^2^)^b^	8.82	<10	Normal (<10)	<8.2	Within 1-1.5-2SD (8.2-9)
	PCR (cm^2^)^a^	0.06	<0.12	Normal (<0.12)	<0.06	Normal (<0.06)
	HLHold (mm)^b^	27.37	<43.3	Normal (<43.3)	<35.4	Normal (<35.4)
	Hant (mm)^a^	7.73	>4.03	Normal (>4.03)	>2.85	Normal (>2.85)
	Hsup (mm)^a^	10.13	>16.92	>2SD Below Mean (<10.17)	>11.36	Within 1-1.5SD (8.38-11.36)
	HL (mm)^a^	2.12	>8.4	>2SD Below Mean (<4.3)	>5.2	Within 1.5-2SD (2.4-1.46)
	Hmax (mm)^a^	12.74	>17.2	Within 1.5-2SD (13.8-10.4)	>10.08	Normal (>10.08)
	HL + Hmax (mm)^a^	14.86	>28.1	>2SD Below Mean (<19.0)	>24.5	>2SD Below Mean (<20.2)

Abbreviations: Hant, anterior hyoid movement; HL, hyoid-to-larynx approximation; HLhold, hyoid-to-larynx approximation at hold; Hmax, maximal hyoid movement; Hsup, superior hyoid movement; PAM, pharyngeal area at maximum constriction; PAH, pharyngeal area at hold; PCR, pharyngeal constriction ratio.

a Indicates values representing 1 cc volume. ^b^Indicates values 20 cc volume.

PAH was normal for male norms but 1.5 to 2 SD above female norms, indicating slightly increased pharyngeal space due to surgical changes. PAM was >2 SD in both norms, while PCR was 1.5 SD below female norms but normal for male norms. HLhold was normal for female norms but >2 SD below male norms, significantly affecting HL and hyoid movement interpretation. Hyolaryngeal measures (Hmax and Hsup) were within normal limits using female norms but abnormal using male norms.

A follow-up VFSS (3 months later) showed a decline in pharyngeal efficiency (DIGEST: 1, S: 0, and E: 1). PAH decreased but remained normal in male norms. PAM declined but was still >2 SD above female norms, while male norms detected improvement. PCR improved per female norms but remained unchanged in male norms. HLhold and HL decreased, with female norms capturing the decline more clearly. Hyoid excursion (Hsup and Hmax) improved but was still considered abnormal using male norms.

For this patient, female norms better captured pharyngeal kinematic changes over time. PAH and HLhold variations highlight anatomical differences, with female norms appearing more sensitive to post-treatment changes. These findings emphasize the importance of considering gender diversity in VFSS interpretation when using kinematic measures to ensure accurate assessment and intervention.

## Discussion

While this study presents only 3 cases of gender-diverse individuals undergoing VFSS assessments, the findings are nonetheless significant given the unique considerations for this underrepresented population. The transgender and gender-diverse community remains vastly understudied in the medical literature. This report serves as an exploratory effort to highlight critical questions regarding how sex-based swallowing norms are applied to transgender and gender-diverse patients—particularly as this patient population continues to grow.

Existing sex-based swallow norms, such as those established by Kendall and Leonard, have documented differences in hyoid displacement, larynx-to-hyoid approximation, PAM, and PAH, yet their application has largely assumed a binary framework of male and female without considering the complexities of gender identity or transition-related care.^2^ Historically, medical literature has often used “sex” and “gender” interchangeably, which may obscure how data were collected and categorized. Additionally, transgender individuals on gender-affirming hormone therapy (GAHT) may not always disclose this treatment due to concerns about stigma, which could impact both clinical assessments and research findings. Misgendering in medical records further compounds these challenges, potentially leading to inappropriate comparisons to sex-based norms and misinterpretation of swallow function.

This study underscores the necessity of accurately documenting a patient’s sex assigned at birth, gender identity, and transition-related medical history—including GAHT use and timing—when evaluating swallowing function. The case of the non-binary patient assessed using female norms due to a lack of documented transition history exemplifies how current clinical practices may overlook the evolving needs of gender-diverse individuals. Given the increasing number of non-binary individuals receiving gender-affirming treatments, it is essential that providers adopt respectful, transparent communication when discussing sex and gender history with patients.

Recognizing the study’s limitations—including the small sample size, lack of comparison groups, and absence of data on transgender men, individuals who transitioned before puberty, and intersex individuals—this report does not seek to establish definitive clinical guidelines. However, it highlights the urgent need for further research into the impact of GAHT and gender-affirming surgeries on swallowing anatomy and physiology. Larger studies will be critical in determining whether current normative data should be adjusted for gender-diverse populations and in refining clinical pathways for evaluating swallowing function in transgender patients.

As clinicians, it is essential to take a holistic and individualized approach when analyzing VFSS studies for gender-diverse individuals. Ethical and sensitive inquiry into gender identity, sex assigned at birth, GAHT use, and the timing of hormonal intervention is necessary to ensure accurate assessment and high-quality care. At our institution, the UCSF Voice and Swallowing Center and Head and Neck Surgical Oncology has adopted an unofficial pathway recommending that patients who begin GAHT after puberty be evaluated using assigned birth sex norms, while those who initiate GAHT before puberty may be assessed using gender-aligned norms. However, further research is required to validate this approach and ensure that it effectively serves the diverse needs of transgender and non-binary individuals.

Furthermore, this exploratory piece serves as an initial step in addressing a critical gap in the literature and important consideration in clinical practice regarding swallowing function in gender-diverse individuals. Given the growing recognition and inclusion of gender-diverse individuals in society, it is important to acknowledge that the increasing number of gender-diverse people may, in part, reflect greater societal acceptance, improved visibility, and the creation of more inclusive and safe spaces where individuals feel empowered to express their identities. Furthermore, as the transgender patient population continues to grow, it is imperative that research efforts keep pace to inform evidence-based, inclusive clinical practices that prioritize the safety and well-being of all patients.

## Conclusion

This commentary highlights the critical need for clinicians to be aware of the potential challenges posed by using sex-matched norms when assessing swallowing physiology in gender-diverse patients. Through the examination of 3 gender-diverse patients at a single institution, we have illustrated the importance of considering individual gender identity and physiological differences, particularly as the number of gender-diverse individuals seeking healthcare continues to rise. As this group remains underrepresented in medical research, these cases serve as an important contribution to the literature and underscore the need for more inclusive, evidence-based practices. Further research is essential to better understand the unique swallowing physiology of gender-diverse patients and to develop norms that are reflective of this diversity. By addressing these gaps, healthcare providers can improve the quality of care and ensure that all patients receive assessments that are both accurate and affirming of their gender identity.
